# Evaluation of patient engagement in medicine development: A multi‐stakeholder framework with metrics

**DOI:** 10.1111/hex.13191

**Published:** 2021-02-24

**Authors:** Lidewij Eva Vat, Teresa Finlay, Paul Robinson, Giorgio Barbareschi, Mathieu Boudes, Ana Maria Diaz Ponce, Michaela Dinboeck, Lukas Eichmann, Elisa Ferrer, Sevgi E. Fruytier, Claudia Hey, Jacqueline E. W. Broerse, Tjerk Jan Schuitmaker‐Warnaar

**Affiliations:** ^1^ Athena Institute Vrije Universiteit Amsterdam Amsterdam The Netherlands; ^2^ Nuffield Department of Primary Care Health Sciences University of Oxford Oxford UK; ^3^ Merck Sharp & Dohme Ltd. Hoddesdon Hertfordshire UK; ^4^ European AIDS Treatment Group (EATG) Dusseldorf Germany; ^5^ European Patients' Forum (EPF) Chaussée d’Etterbeek Brussels Belgium; ^6^ Alzheimer Europe Luxembourg Luxembourg; ^7^ Novartis Pharma AG Basel Switzerland; ^8^ Novo Nordisk A/S Bagsvaerd Denmark; ^9^ EURORDIS‐Rare Diseases Europe Paris France; ^10^ Merck Healthcare KGaA Darmstadt Germany

**Keywords:** impact, metrics, monitoring and evaluation, patient engagement, patient participation, quality indicators

## Abstract

**Background:**

Patient engagement is becoming more customary in medicine development. However, embedding it in organizational decision‐making remains challenging, partly due to lack of agreement on its value and the means to evaluate it. The objective of this project was to develop a monitoring and evaluation framework, with metrics, to demonstrate impact and enhance learning.

**Methods:**

A consortium of five patient groups, 15 biopharmaceutical companies and two academic groups iteratively created a framework in a multi‐phase participatory process, including analysis of its application in 24 cases.

**Results:**

The framework includes six components, with 87 metrics and 15 context factors distributed among (sub)components: (a) Input: expectations, preparations, resources, representativeness of stakeholders; (b) Activities/process: structure, management, interactions, satisfaction; (c) Learnings and changes; (d) Impacts: research relevance, study ethics and inclusiveness, study quality and efficiency, quality of evidence and uptake of products, empowerment, reputation and trust, embedding of patient engagement; (e) Context: policy, institutional, community, decision‐making contextual factors. Case study findings show a wide variation in use of metrics. There is no ‘one size fits all’ set of metrics appropriate for every initiative or organization. Presented sample sets of metrics can be tailored to individual situations.

**Conclusion:**

Introducing change into any process is best done when the value of that change is clear. This framework allows participants to select what metrics they value and assess to what extent patient engagement has contributed.

**Patient contribution:**

Five patient groups were involved in all phases of the study (design, conduct, interpretation of data) and in writing the manuscript.

## 1 INTRODUCTION

In the past decade, patients, industry, regulators, researchers and health professionals have identified opportunities to improve the medicine development process. Firstly, studies have shown that there is sometimes a mismatch between the research priorities of patients and the research that is conducted by academia and the pharmaceutical industry.^1^, ^2^, ^3^ As a result, some new medicines and therapeutic interventions that enter the market are perceived to have little or no added value for patients.^2^ Secondly, the design of studies is not always optimized for the experience of study participants.^4^, ^5^ This can result in unnecessary burden for patients, avoidable protocol amendments and a delay in access to new medicines and technologies.^4^, ^6^ Thirdly, the transparency of studies can be improved by making positive as well as negative findings accessible to those who need to make decisions about their own health.^5^ Finally, clinical trials focussing solely on the evidence required for regulatory approval often lack patient‐relevant outcome measures. Medicines and technologies may therefore enter the market without a full understanding of the benefits for patients.^7^

Evidence suggests that engaging patients in medicine development results in studies that align better with patients’ needs and benefit from enhanced performance in terms of efficiency and quality.^8^, ^9^, ^10^, ^11^ Pharmaceutical companies and researchers across the world continue to expand their efforts to engage patients in research and development (R&D). Patient engagement is slowly becoming more common. However, embedding and systematizing it in organizational decision‐making remains challenging, partly due to lack of agreement on its value and the means to evaluate it.^12^, ^13^ There is a tension between its intrinsic value, reflecting a democratic approach of fairness, transparency and accountability (‘nothing about us without us’^14^) and an instrumental approach referring to patient engagement as a means to improving the quality of research.^15^ Indeed, some argue that patient engagement cannot be seen as an intervention to be evaluated, but that it is a prerequisite for a people‐centred health‐care system.^16^ Patient engagement may best be described as a process of knowledge exchange needed to better integrate patient perspectives, needs and priorities^17^ rather than a typical intervention, which requires a different evaluation approach.

A recent literature review shows that some metrics and methods are available, but that these are not sufficient to understand (a) the mechanisms to impact, nor (b) whether the interaction between researchers and patients leads to a culture change.^18^ Previous studies have identified a number of challenges to assessing the impact of patient engagement, such as the lack of well‐defined endpoints, the delayed nature of impact, the absence of reliable measurement tools and accepted criteria for judging the success of engagement.^18^, ^19^ The value of patient engagement can vary by stakeholder's perspectives and therefore the measures of interest will differ accordingly.^20^

Numerous frameworks provide guidance for undertaking patient engagement. Far fewer support evaluation of engagement; those that do outline concepts rather than provide detailed operational guidance.^21^, ^22^ Research suggests that co‐designed evaluation frameworks are most likely to be locally relevant and used in practice but these tend to be context‐specific and may be difficult to apply to different initiatives.^22^ The PARADIGM consortium, a public–private multi‐stakeholder partnership co‐led by the European Patients’ Forum and The European Federation of Pharmaceutical Industries and Associations (EFPIA), aimed to develop a framework to support collaborative evaluation of patient engagement in the field of medicine development. Our preparatory literature review indicated that an evaluation framework required to show the return on patient engagement from all stakeholders’ perspectives, had not previously been developed. In common with Boivin et al,^23^ we required such a tool to be evidence based, to encompass all stakeholders’ perspectives and to be comprehensive and user‐friendly.

Accordingly, the aim of this research project was to co‐design a framework for monitoring and evaluating patient engagement initiatives, in order to support meaningful engagement in medicine development. Our research combined the perspectives of patient organizations, industry, academics, regulators and Health Technology Assessment (HTA) bodies. We focused on three decision points at which the patient perspective is likely to be valuable: research priority‐setting, design of clinical trials and early dialogues with regulators and HTA bodies. This paper presents the conclusions of this work: a co‐designed evaluation framework which can be used to assess the quality and impact of patient engagement in medicine development for all stakeholder groups.

## 2 THEORETICAL BACKGROUND

Effective change in health research and its evaluation can be difficult to achieve, particularly when numerous stakeholder groups are involved. Each group will participate in the endeavour with their own social and professional drivers, their previous experiences and individuals’ associated instinct in approaching this type of initiative. Few successful change initiatives are achieved solely by instinct, not least because personal intuition and recall tend to be cognitively biased.^24^ The application of theory enables illumination and understanding of procedures and how they can be successfully changed, as demonstrated by evaluation that illustrates their impact. Theory can ensure the rigour needed to effect and evaluate change in a way that the learning and experience it provides is transferable and can be embedded in future initiatives.^25^ Therefore, because we sought to develop a scientifically robust evaluation framework for the change to sustainable patient engagement, we chose to adopt a combination of theoretical approaches to inform this project.

We considered various evaluation approaches on which to base our framework including those shown in Table 1 and briefly discuss the applicability of these approaches for evaluating patient engagement. Some studies of patient engagement use impact evaluation; for most of these, it remains unclear how impact was (or was not) reached. Impact evaluation cannot easily be used to evaluate patient engagement initiatives^26^ due to the varied approaches to patient engagement and the varied contexts in which it takes place. Furthermore, the impact of patient engagement is influenced by multiple factors. We argue that the impact of patient engagement can best be determined by applying a set of linked measures.^18^ Theory‐based evaluation approaches (eg programme evaluation, realist evaluation)^27^ have been recommended for evaluating patient engagement,^28^, ^29^ as these approaches also focus on the conditions necessary for effectiveness.^25^, ^27^ Therefore, we drew on theory‐based evaluation approaches for the development of the monitoring and evaluation (M&E) framework. Although the primary objective of the project was to develop a framework to demonstrate the ‘return on the engagement’ for all players, we decided to also include metrics that assess whether or not the conditions for reaching the intended impacts are in place to stimulate reflection and continuous improvement of engagement practices. For example, we adopted the importance of context in evaluating outcomes from a realist evaluation approach. Furthermore, we used a logic model approach, often used in programme evaluations, to identify metrics that relate to each other (set of metrics). A logic model explains how activities are understood to contribute to a chain of results that produce impacts. In addition, we involved all stakeholders in the framework development process to ensure identification of a wide variety of impact metrics (eg impacts for research, people and organizations).

**TABLE 1:** Evaluation approaches (adapted from: Pawson and Tilley 1997^46^; Blamey and Mackenzie 2007^27^; Westhorp 2014^47^; Wong et al 2013^48^; Rogers 2008^49^; BetterEvaluation^50^)

Evaluation approach	Evaluation question	Examples of study designs	Applicability
Impact evaluation	What is the effect of the intervention? Does ‘it’ work?	Experimental design Quasi‐experimental design Comparative case study design	This approach is suitable for evaluating singular interventions where there is a direct causal influence of the intervention, in a setting that can be controlled (to reduce confounding) and in which one group can be compared to another.
Programme evaluation	Is the programme or initiative designed in a way it can achieve its intended outcomes?	Descriptive study design	This approach is suitable for evaluating complex interventions, in a setting where a series of outcomes lead to the final impacts.
Realist evaluation	‘What works in which circumstances and for whom?’	Single or multiple case study design	This approach is appropriate for evaluating initiatives or programmes that seem to work but where ‘why’, ‘when’, ‘how’ and/or ‘for whom’ is not yet understood, in a setting where the context influences how an intervention is implemented and how actors respond to it (or not).

## 3 METHODS

This project was conducted within the context of the IMI‐PARADIGM (Innovative Medicines Initiative‐Patients Active in Research and Dialogues for an Improved Generation of Medicines) project. We created a multi‐stakeholder working group consisting of representatives of five European patient groups, 15 biopharmaceutical companies and two academic institutions. This working group was tasked with:

- Reviewing the literature on M&E of patient engagement
- Development and testing of a M&E framework
- Identification and selection of appropriate metrics for M&E
- Clarification of terminology and language to be used.

The working group was involved in all phases of the project (as described below). They provided feedback on documents and versions of the framework, co‐analysed case study data, were involved in writing publications and in other dissemination activities. All working group members had a say in the framework development process by participation in monthly working group calls, workshops, polling/voting tools and by providing written feedback. The researchers facilitated the decision‐making process by providing options, draft documents, questions, theoretical guidance and overall coordination of the research.

### 3.1 Terminology

A variety of terminology is used in the literature. In Table 2, we provide an overview of terms and definitions used by the authors throughout this paper.

**TABLE 2:** Definitions

Concept	Description
Patient engagement	The effective and active collaboration of patients, patient advocates, patient representatives and/or carers in the processes and decisions within the medicines lifecycle, along with all other relevant stakeholders when appropriate.^51^ This may include activities at specific decision points and/or on‐going collaborations throughout the R&D cycle.
Monitoring	The formative evaluation of patient engagement practices in order to strengthen these practices.
Evaluation	The ‘systematic acquisition and assessment of information to provide useful feedback about …’ patient engagement practices.^52^ Summative evaluation examines the effects of patient engagement practices on various measures including outcomes, impact and cost‐benefit.
Value	The benefits of patient engagement (in relation to the direct and indirect costs) for individuals and organizations (thereby acknowledging that value can have different meanings to different people, for example value for money, value for time, value for health).
Research priority‐setting	Any process aimed at constructing priorities or agendas for health research and medicine development, that raises awareness and change the way research funding is allocated.
Design of clinical trials	Any process aimed at the development or design of clinical trials for medicine development at any stage.
Early dialogues with regulators and Health Technology Assessment (HTA) bodies	Any process in which medical technology developers communicate with regulatory bodies and/or HTA bodies prior to health technology assessment. Early dialogue can happen only with regulators (eg scientific advice), jointly with regulators and HTA bodies (to discuss data requirements to support decision‐making on marketing authorization and reimbursement simultaneously) or only with HTA bodies (eg EUnetHTA multi‐HTA dialogues)
Components	By components, we mean dimensions or criteria used for monitoring and evaluation, which need to be translated into measures called ‘indicators’ or ‘metrics’, which may be quantitative and/or qualitative. By subcomponents, we mean categories with metrics related to a specific component.
Set of metrics	An agreed group of metrics that relate to each other and align to a certain context, with methods/tools to collect the information.
‘Forward’ and ‘backward’ metrics	By ‘forward’ and ‘backward’, we mean metrics that link to the given metric and would be measured before or after the given metric is measured.
Linked metrics	By ‘linked’ or ‘related’, we mean additional metrics that complement the given metric. Metrics which are relevant to measure in combination with other metrics (eg to improve understanding of how impact can be reached).
Short and long‐term results	With short‐term results, we mean outcomes that can be measured directly after engagement activities. With long‐term results, we mean impacts that become evident in the years after engagement activities. This could be during or after an active collaboration or research study.
Reflexivity	The capacity to reflect upon (social) practices, assumptions, beliefs and values and to challenge and change those that are undesirable through enquiry, dialogue and learning.

### 3.2 Study design and approach

**FIGURE 1:**
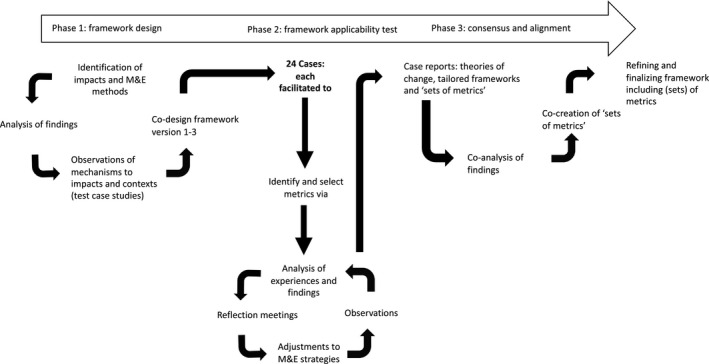
Study design and participatory action research process

We used participatory action research (see Figure 1) to develop and refine the M&E framework, as described by Van Mierlo et al 2010.^30^ The researchers’ role was to partner with companies and patient organizations to facilitate discussions and support early attempts to monitor and evaluate their patient engagement initiatives. The process included three distinct phases: (1) design phase, (2) testing phase and (3) consensus and alignment phase. Each of these involved a different methodological approach described below.

#### 3.2.1 Phase 1: Framework design

The aim of the design phase was to develop early versions of the M&E framework by identifying (a) impacts reported from historic patient engagement initiatives, (b) conditions needed to achieve this impact, and (c) suggested metrics for M&E of patient engagement. As part of this, a literature review was conducted. In addition, we identified and analysed existing M&E frameworks in the field of patient engagement. The methods and results of the literature review have been published in a separate paper.^18^ While this project focused on three specific decision‐making points in medicine development, the literature review study used broader search limits to capture related publications in other areas of health research that were applicable.

Three key informant interviews were conducted (LV) to gather experiences of M&E of patient engagement and possible metrics. In addition, researchers conducted six test case studies with PARADIGM partners to gather insights into the conditions needed to achieve impact and contextual factors that may influence the impact of patient engagement. Interviews were conducted with patient engagement leads of pharmaceutical companies and patient organizations and combined with an analysis of engagement‐related documents (including agendas, minutes, outcome reports).

Informed by the work above, an early version of the framework was developed by researchers (LV, JB, TF). This was reviewed by a small group of PARADIGM partners; feedback from the multi‐stakeholder working group was then sought and resulted in version three, which entered the test phase.

#### 3.2.2 Phase 2: Framework applicability test

The aim of the test phase was to apply the draft framework to real‐world patient engagement initiatives in the process of medicine development. We used the M&E framework to validate the identified (sub)components and to select and test suggested metrics in practice. Our approach to this was to use a case study design (Yin 2018)^31^ in which researchers each worked with separate cases on Phase 2 of the study (see Figure 1).

##### Participant case studies

Partners of the IMI‐PARADIGM consortium agreed to contribute and participate with case studies of patient engagement initiatives in medicine development. In total, 24 patient engagement initiatives were included as cases (see Table 3).

**TABLE 3:** Types of cases contributed to the framework applicability test phase

	Nr of cases (n = 24)
Initiated by	‐ Patient organization: 6 ‐ Industry: 14 ‐ Regulatory bodies: 4
Decision points	‐ Research priority‐setting: 2 ‐ Design of clinical trials: 13 ‐ Early dialogue: 1 ‐ Other (eg HTA, data analysis, dissemination): 4 ‐ Multiple decision points: 4
Timeline	‐ Underway/on‐going: 10 ‐ Completed < 1 year ago: 7 ‐ Completed > 1 year ago: 1 ‐ Generic (not a specific timeline, organizational level): 6

##### Data collection

**FIGURE 2:**
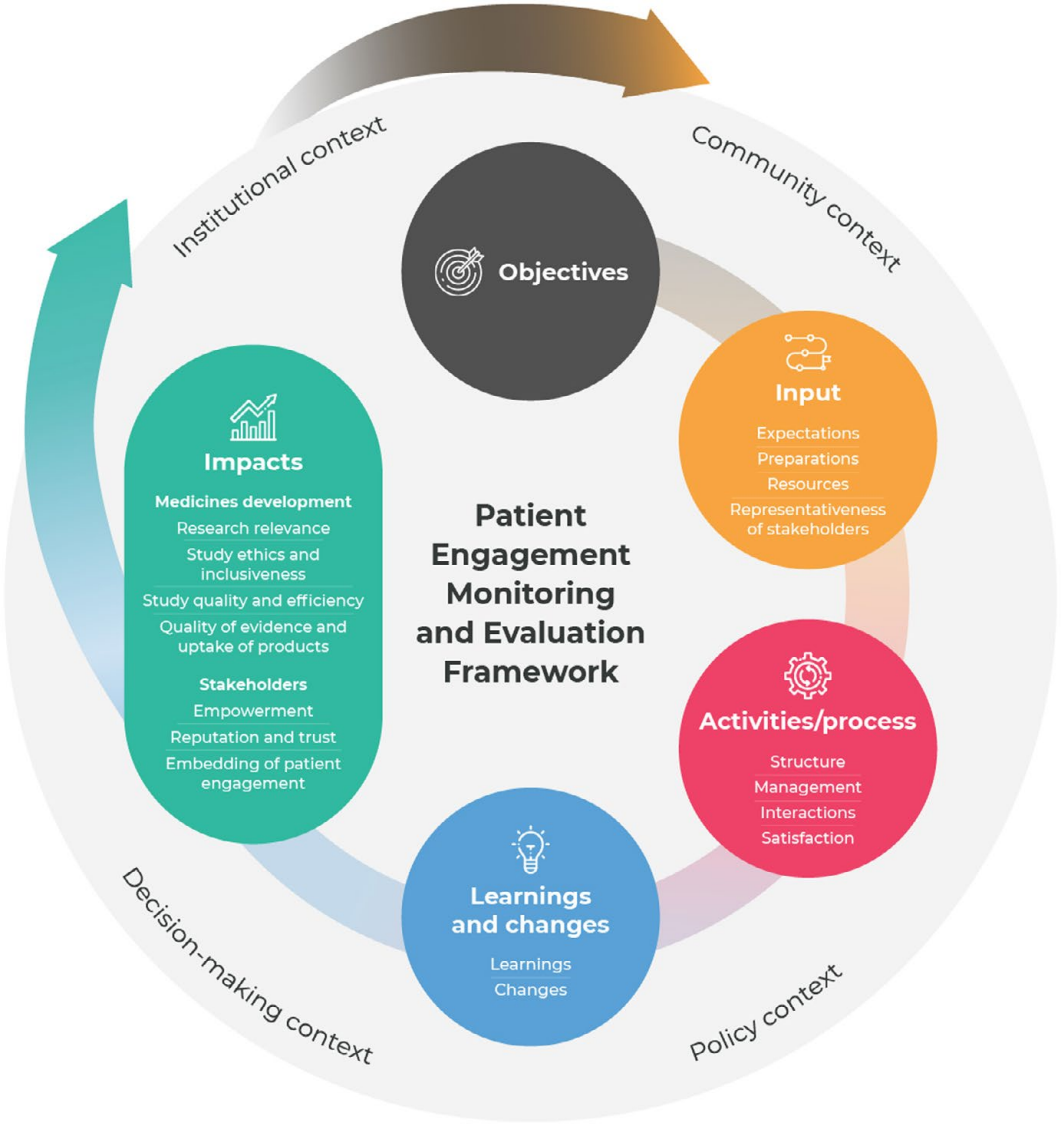
Patient Engagement Monitoring and Evaluation framework

Applicability test data were collected between June 2019 and May 2020. Case study contributors were asked to describe their patient engagement initiative per component of the M&E framework (see Figure 2). Furthermore, they were asked to select appropriate metrics per component of the framework for M&E of their initiative(s). They could select metrics identified in the design phase, metrics they were already measuring or suggest new metrics. Their input was analysed by their assigned member of the research team (LV, TF, NG, CG, SF, LD or TS). Reflection meetings were held between the researchers and the case contributors to discuss the framework, metrics and their applicability. A tailored ‘set of metrics’ for each case was developed using an iterative approach. Cases were coded to ensure anonymity and data were stored by the research team on the VU University's encrypted platform.

##### Data analysis

A descriptive, qualitative approach was used to analyse the data per component of the framework in a cross‐case analysis.^31^ A case report was sent to each case for discussion and validation. The research team co‐analysed the metrics from all cases; discrepancies were discussed and similar metrics were merged. Next, the working group members involved in writing this article co‐analysed anonymized case data. Lessons learned from the cases for framework and metrics development were discussed. Furthermore, the applicability of the framework was reviewed and any changes that derived from the case studies were discussed during the consensus and alignment phase.

#### 3.2.3 Phase 3: Framework consensus and alignment

The aim of the consensus and alignment phase was to develop agreed ‘sets of metrics’. An online consensus‐building workshop was held with all stakeholder groups. All working group members were invited (n = 32 participated). It was agreed that ‘sets of metrics’ would be created per patient engagement objective as identified in the case studies. Participants prepared in advance by reading a document with possible criteria for metrics selection (see Appendix S1). In small break‐out groups, participants worked on the creation of a set of metrics by prioritizing and selecting metrics per framework component from the full lists of metrics that were identified in the previous phases. This process was facilitated by the researchers (LV, TF, CG, SF, LD, TS, LH, NF). Possible measurement methods were identified from the literature review and the case studies’ own methods and were mapped to the sets of metrics. Monthly calls were held with the working group members to reach consensus on the final framework, including (sub)components, (sets of) metrics and measurement methods.

## 4 RESULTS

The framework (Figure 2) includes (sub)components and metrics to assess the quality and impacts of patient engagement. Below, we describe each component of the framework, discussing the metrics and underlying logic using examples from case studies. An overview of all the metrics categorized to each (sub)component of the framework can be found in Appendix S2, including a description of each metric, related metrics and possible methods for measurement. All metrics are formulated neutrally as results can be positive (benefits) or negative (direct and indirect costs and challenges). Examples of quotes illustrating the metrics can be found in Appendix S7.

### 4.1 Objectives of patient engagement

Every initiative is unique and has its own objectives describing the aims the initiative intends to achieve. Impacts are related to objectives in terms of the intended long‐term results achieved. However, impacts could also include unintended consequences which are important to elicit. Seven overarching objectives of patient engagement were derived from the case studies:

- Improved experience of patient participation in clinical trials/medicines research
- Improved quality of evidence generation
- Knowledge exchange/knowledge gain
- Access to clinical trials and medicines
- Improved transparency, trust and relationships
- Improved efficiency of the medicine development process
- Alignment of product development with (unmet) patient needs

### 4.2 Input

Input indicates whether or not the conditions for meaningful and sustainable patient engagement are in place. The 13 input metrics (see Appendix S2) developed were grouped into four subcomponents for monitoring. Below, we describe some examples of what case study participants measured, starting with the subcomponent Expectations, followed by Preparations, Resources and Representativeness of stakeholders.

#### 4.2.1 Expectations

This subcomponent includes metrics that assess the needs, beliefs and priorities of those involved in patient engagement. Preparations and expectations were mentioned as predictive variables for successful patient engagement by case study participants. For example, four cases captured the needs and priorities of stakeholders. Knowing needs/priorities helps define relevant issues to be addressed and reduces the risk of tokenism and ‘confirmation bias’. Taking the needs and priorities of all stakeholders into account enhances satisfaction and recommendations made, indirectly influencing learnings, changes and impacts.

#### 4.2.2 Preparations

This subcomponent includes metrics that assess the accessibility and preparedness for patient engagement. It is believed that those who are well prepared can participate more effectively, resulting in better outcomes. Therefore, some cases (n = 4) decided to monitor the accessibility of preparatory materials and/or feeling of preparedness of those involved.

#### 4.2.3 Resources

This subcomponent includes metrics that assess the material, human and financial resources used to carry out a patient engagement initiative. Dedicated time and funding are needed to ensure meaningful and sustainable patient engagement. Numerous cases (n = 17) selected the metric ‘money spent’ (eg reimbursement, compensation) and ‘time spent’ (n = 10) commonly measured in hours or full‐time equivalent breakdown.

#### 4.2.4 Representativeness of stakeholders

This subcomponent includes metrics that assess the expertise and diversity of individuals involved. Carefully selecting patient partners is seen as a critical step to obtaining meaningful patient insights. The diversity of patient representatives is seen as a predictor for the diversity of learnings and recommendations made. Many cases (n = 15) selected this metric. This includes demographic diversity as well as disease state, treatment experience, experience of patient engagement or involvement in clinical studies. Fewer cases (n = 6) decided to track the diversity of staff/departments representatives. This can give an indication of expertise/responsibilities of those involved which may link to the number of patient recommendations implemented and the embedding of patient engagement.

### 4.3 Activities/Process

The process gives an indication of how the implementation of patient engagement is progressing and can elucidate areas for improvement. The 16 process metrics developed (see Appendix S2) were grouped into four subcomponents for monitoring. Below, we describe some examples of what case study participants measured, starting with the subcomponent Structure, followed by Management, Interactions and Satisfaction.

#### 4.3.1 Structure

By structure, we refer to the way the patient engagement activities are organized. This subcomponent includes metrics that describe the general characteristics such as ‘what took place’ and ‘when’. For example, the metric ‘number, type and frequency of engagement activities’ was selected by many cases (n = 13). This could be tracked at organizational or trial level and can indicate how embedded patient engagement is. Some cases (n = 4) selected the metric ‘timing of patient engagement activities with stages of the R&D cycle’. This could be tracked per research stage (eg priority‐setting, pre‐clinical development, phase I, phase II etc) and/or at specific time‐points in designing clinical studies, such as ‘target product profile’, ‘clinical development plan’, ‘protocol concept’, ‘protocol optimization’. Monitoring this helps to understand when best to engage patients and why certain impacts may not be achieved, for example due to a lack of or limited engagement at certain decision points.

#### 4.3.2 Management

By management, we refer to how engagement activities are facilitated, for example, metrics that assess ‘satisfaction with the moderation’ or ‘satisfaction with support from activity organisers’. These metrics help organizers improve the facilitation of patient engagement activities, and thereby indirectly the impacts.

#### 4.3.3 Interactions

With interactions, we are referring to the quality of interactions during the process, for example metrics that assess ‘feelings of trust’, ‘honesty’, ‘transparency’, ‘respect’, ‘give and/or take relationship’ and ‘opportunity to contribute’, factors that indirectly influence learnings, changes and impacts.

#### 4.3.4 Satisfaction

By satisfaction, we refer to the overall experience of those involved with the engagement process. Measuring satisfaction with patient engagement activities is seen as important by most cases (n = 17) as this is considered a predictor for willingness to continue collaboration and the overall value of patient engagement.

### 4.4 Learnings and changes

Learnings and changes are the short‐term, direct results of patient engagement activities which give an indication of the progress made towards impacts. It should be noted that ‘changes’ may imply that there was an original research idea that changed after the engagement of patients. This may not always be the case: changes to original ideas may not occur where they are co‐created and emerge collaboratively during engagement activities. The 13 metrics (see Appendix S2) developed were grouped into two subcomponents for evaluation. Below, we describe some examples of what case study participants measured, starting with the subcomponent Learnings followed by Changes.

#### 4.4.1 Learnings

Learnings refer to the insights and recommendations people take‐away from the patient engagement activities. Measuring learnings is seen as important by multiple cases (n = 14). Learnings relate to industry learning about patients' experiences of a condition/technology, and patient advisors learning about processes involved in medicine development. However, it can be hard‐to‐define specific metrics for learnings upfront, as at that time those involved may not know what they do not know.

#### 4.4.2 Changes

This subcomponent includes metrics that capture changes to research (such as altered research questions, design, processes, outcome measures), or changes to individuals (such as changes in attitudes). The metric ‘number and type of actions and recommendations implemented’ was selected by multiple cases (n = 15). These differed widely, influenced by stakeholders’ priorities for engagement, the diversity of those involved, the timing of engagement and the context. It can also be insightful to track the reasons for (not) implementing a particular action or recommendation, leading to a better understanding of inhibiting factors the patient engagement initiative is facing. Changes in perceptions or attitudes could be shown by comparing results before and after patient engagement activities and may reveal incorrect assumptions. This is seen as important in contexts where patient engagement is not embedded in the organizational culture, in order to encourage people to acknowledge the value of patient engagement.

Learnings and changes metrics could also be tailored to a patient engagement initiative. For example, the objective of Case 3 was to get access to medicines for people co‐infected with two viruses; they tailored metrics to specific learnings, for example ‘awareness of co‐infected people's situation’ and specific changes, for example ‘number of clinical trials changing inclusion and exclusion criteria’.

### 4.5 Impacts

If the acquired learnings are put into practice, then long‐term impacts for medicine development and stakeholders may become tangible. Impact metrics (see Appendix S2) differed widely by case, depending on the objectives of patient engagement. Results can be positive or negative, intended and unintended. There can be overlap between ‘learnings and changes’ and ‘impacts’, some metrics may fit in both components or may be a short‐term result for one collaboration while being a long‐term result for another collaboration. For example, changes in research priorities and goals may arise immediately after engagement, but could also emerge in the years after engagement activities. The 45 developed impact metrics were grouped into seven subcomponents for evaluation. Below, we describe some examples of what case study participants measured, starting with the impact metrics related to medicine development, followed by metrics that assess the impacts for stakeholders.

#### 4.5.1 Research relevance

This subcomponent includes metrics that assess the relevance of study areas, topics and questions to patient needs. Mostly cases that involved patients in research priority‐setting selected metrics related to the research relevance, for example the ‘emergence of new research areas’ and ‘relevance of new studies to patients’ needs’.

#### 4.5.2 Study ethics and inclusiveness

This subcomponent includes metrics that assess the diversity and accessibility of studies. For example, some patients suggested assessing the impact on inequalities in research, such as the ‘number of trials including under‐represented groups’.

#### 4.5.3 Study quality and efficiency

This subcomponent includes metrics that assess the speed of studies and influencing factors such as study participants’ experiences of trials. A number of cases selected metrics related to study quality and efficiency as their overall objective is to improve the efficiency of medicines R&D. Some cases tried to make a comparison between enrolment rates of trials that engaged patients in the planning phase against expected rates for the specific trial, or tried to compare trial sites that incorporated patient recommendations and those that did not implement this recommendation within the same trial. Various contextual factors influence trial efficiency (including ethics, regulations, trial sites); therefore, it is argued that efficiency cannot unequivocally be linked to patient engagement. However, study participants’ experience is seen as a predictor for the efficiency of trials by cases. The experience of study participants, in turn, may depend on the level of influence patients had on the protocol. Some cases also selected metrics which can be seen as predictors for recruitment rates, such as patients’ understanding/accessibility of information materials and consent form or patients’ willingness to participate in clinical trials.

#### 4.5.4 Quality of evidence and uptake of products

This subcomponent includes metrics that assess the quality and availability information used for decision‐making by regulatory bodies and patient communities as well as the accessibility and usability of products. For example, industry‐led cases which involved patients in early dialogues with regulators selected metrics related to the quality of evidence such as ‘percentages of trials with patient‐reported outcomes measures’ and ‘degree to which patient engagement will help to demonstrate the value of a product to regulatory bodies’. Regulatory bodies seemed interested in measuring ‘the quality of the feedback provided by patients’ and ‘the quality of scientific advice provided by regulators/HTA in terms of relevance to patients’ needs’.

#### 4.5.5 Empowerment

This subcomponent includes metrics that capture the process of becoming more confident and shifts in decision‐making authority or power. Patients often valued metrics related to empowerment such as ‘feeling of being heard’, ‘feeling of contributing to research and the patient community’ and ‘quality of life as a result of engagement’. Metrics also differed between patient groups. For example, young people were interested in measuring personal and professional skills gained from engagement, while people with Alzheimer's disease suggested measuring the ‘degree to which patient engagement activities help reduce stigma’.

#### 4.5.6 Reputation and trust

This subcomponent includes metrics that capture beliefs and opinions about working relationships, research, a company, organization and/or community, for example ‘feelings of trust’ and ‘level of confidence in research’.

#### 4.5.7 Embedding of patient engagement

This subcomponent includes metrics that capture wider consideration of patient engagement in R&D and organizational decision‐making, for example ‘number of industry research teams engaging patients’. This can be used to demonstrate cultural change within companies, indicating the extent to which patient engagement becomes customary in an organization.

### 4.6 Context

The impact of patient engagement cannot be determined without considering context. Indeed, case study findings suggest that contextual factors influence the success of patient engagement initiatives, and that successful patient engagement initiatives influence the context. The four context components—policy, institutional, community, decision‐making—were based on literature^1^ and confirmed in stakeholder discussions; 15 contextual factors (see Appendix S2) were identified in the case studies. Below, we provide four examples of contextual factors (see Box 1).

Box 1 **Examples of contextual factors**

**Policy context factor (Case study 3)**

There were no regulatory requirements or recommendations for studying therapies in people living with two different viral infections affecting their health at the same time (co‐infection); co‐infection was only viewed as a risk factor for those people to participate into clinical trials. Therefore, there was no incentive for industry to provide access to clinical trials for co‐infected people, decreasing the chance of implementing patient recommendations related to access issues. (barrier).

**Institutional context factor (Case study 17)**

The company has a long history of patient engagement for [disease] and is therefore familiar with the common issues faced by patients in this therapeutic area. Consequently, the learnings and changes, and potentially the impact of the patient engagement initiative may be modest. (barrier).

**Decision‐making context factor (Case study 14)**

The trial protocol was already approved when discussing potential recruitment challenges and retention with patients; therefore, patients had little influence on the study focus, study population, design of the trial etc Thus, the impact on study participant experience and ultimately the efficiency of the trial, may be limited. (barrier).

**Community context factor (Case study 6)**

There is a well‐established European patient organization for involvement of patients with [disease] in the drug development process. This facilitates (early) access to a diverse group of patients, influencing the potential learning, changes and impacts. (enabler).

It should be noted that contextual factors can also influence M&E strategies. For example, in Case 14, the team tried to look at avoidable errors, using the study of Levitan et al (2018)^32^ as an example. The culture within the company was perceived as a barrier to measuring avoidable errors as people dislike reflecting on what went wrong. Therefore, measuring the number of avoidable protocol amendments was seen as a meaningful metric for some, but not feasible within all companies.

### 4.7 Developing a tailored framework

The wide variation in selected metrics per component of the framework shows how difficult it is to uniformly define or standardize metrics for evaluation of patient engagement. Therefore, we recommend developing a M&E strategy that is tailored to the patient engagement initiative. The framework can best be tailored by following the steps below (see Box 2 for an example).

1. Stakeholders decide on the objectives for patient engagement and agree on the purpose of M&E, taking the context into account.
2. Stakeholders develop a theory of change, using the framework, to show the expected path from input to impact by specifying the input and activities that should result in learnings and changes to achieve the intended objectives. Stakeholders select metrics that address their information needs for each component of the framework. (Sample sets of metrics can be found in Appendix S2 and selection criteria can be found in Appendix S1.)
3. Stakeholders identify qualitative and quantitative methods to collect information and develop a monitoring and evaluation plan. (Sample questions, methods and tools can be found in the Appendices S3, S4, S5 and S6 of this paper.)
4. A feedback loop is established for all stakeholders (eg following Mierlo et al 2010^30^). Data analysis and reflection on short‐term results should be repeatedly undertaken to adapt the initiative and enhance learnings that set the stage for achieving impacts. Stakeholders should also consider contextual factors that may facilitate or hinder the success of initiative. (Possible context factors can be found in Appendix S2 of this paper.)

Box 2 **A case example**

**Background**

This case was initiated by a pharmaceutical company, bringing together industry staff and patients to discuss issues regarding patient experience in clinical trials.

**Step 1: setting objectives**

The objective of the case was to improve experiences of patients participating in clinical trials to ultimately speed up medicines’ development. The purpose of M&E was to 1) prioritize patient engagement activities in order to spend the available resources wisely, 2) learn about and educate colleagues on when and how patient engagement adds value to clinical trials, 3) provide feedback about learnings and the resulting outcomes and impact on those involved and 4) improve collaborations and other patient engagement activities.

**Steps 2 and 3: theory of change, tailored set of metrics with methods/tools**

There is a burden for patients who participate in clinical trials (context). [Company] has chosen to engage patients as it is believed it can make the trials more patient‐friendly (input). Through patient advisory board meetings, [company] gains insight into patient needs (activities/process). Patients provide input for trial design decisions both before and after protocol approval for example on visit structure, frequency, materials, outcome measures. The activities lead to new insights (eg about patient experiences) and changes in trial designs (learnings and changes), which lead to satisfactory patient experiences in trials, higher recruitment and retention in comparison against expected for the clinical trial (impacts), and therefore faster approval (overall objective).

The initial M&E plan focussed heavily on measuring impact. While data from the Study Participant Feedback Questionnaire (SPFQ) can evaluate whether the initiative reaches its objectives, monitoring proxy metrics give insight into whether or not the initiative is working the way it is intended and whether the acquired insights are put into practice to achieve impacts. By using the framework, the initiative created a tailored set of metrics (see Table 4). A pre‐ and post‐meeting survey was developed by the company to collect information from industry staff and patients involved. Other methods are also used including interviews and logbooks.

**Step 4: reflexive monitoring**

A feedback loop has been created. The outcomes of each patient advisory board meeting are discussed with the research team (eg learnings and expected actions), then followed up every six months to continuously track the implementation of these actions. The patients who participated receive an outcome report summary directly after the meeting and an update about the actions implemented every six months. The company plans to implement the SPFQ survey at the start, during and at the end of trials. The survey may need to be adjusted to link the study participant experiences to specific changes made based on patients’ input. The M&E results have been used to educate colleagues, inform meeting agendas and enhance the patient engagement initiative.

**Reflection on the application of the framework and its value**

The industry partners selected metrics during an interactive in‐company workshop. The application of the framework could have been enhanced by engaging patients in the development of the theory of change, selection of metrics and development of measurement tools. The framework helped to consider metrics the team had not thought of, such as metrics that can be used to track progress made.

**TABLE 4:** Tailored set of metrics

Framework component	Subcomponent	Metrics/factors	Methods/tools
Input	Expectations	Expectations of the meeting	Pre‐meeting survey
	Representativeness of stakeholders	Type/level of knowledge of patients and staff involved	Pre‐meeting survey
		Diversity of patients/staff/departments involved	Logbook
	Preparations	Quality and accessibility of information	Post‐meeting survey
	Resources	Time and money spent by each stakeholder	Logbook
Activities/Process	Structure	Number of patient advisory board meetings	Logbook
		Timing of engagement with stages of the R&D cycle	Logbook
		Topics discussed	Meeting agenda
	Management	Clarity of the goals	Post‐meeting survey Pre‐ and post‐meeting interview
	Interactions	Opportunity to contribute to the discussion	Interview
	Satisfaction	Satisfaction with participation in meetings	Post‐meeting survey Post‐meeting interview
		Satisfaction with fulfilling patient engagement activity's expectations	Post‐meeting survey
Learnings and changes	Learnings	Number and type of insights, actions and recommendations	Outcome report
		Awareness and knowledge about patients’ needs among staff	Post‐meeting survey Post‐meeting interview
		Patients’ knowledge about clinical trials	Post‐meeting survey Post‐meeting interview
	Changes	Changes to research protocols	Outcome report Post‐meeting survey
		Expected influence of patients' insights on research decision‐making	Post‐meeting survey
Impacts	Study quality and efficiency	Study participant experience in trials	Study Participant Feedback Questionnaire (SPFQ) Exit interviews
		(Expected) recruitment and retention rates	Post‐meeting survey Log sheet trial sites
		Patient adherence and compliance rate to study procedures	Log sheet trial sites
	Reputation and trust	Confidence in research	Post‐meeting survey Post‐meeting interviews
Contextual factors	Policy	Conflict of interest policies	Reflection session
	Institutional	Company's experience in a disease area	Reflection session
		Organizational culture and commitment to patient engagement	Reflection session
	Decision‐making	Timing of engagement with respect to decision‐making	Reflection session
	Community	Availability of patient groups	Reflection session

### 4.8 Reflections from the case studies

Our case study findings show that stakeholders struggled to select meaningful metrics for their initiative and/or felt overwhelmed by the number of possible metrics. Therefore, during a multi‐stakeholder workshop, participants co‐created *sets of metrics*, to provide examples of how metrics can be logically linked based on the patient engagement objective. Four of the seven identified objectives were selected for the first ‘set’ creation workshop. By a ‘set of metrics’, we mean a group of metrics that evaluate each component of the Framework, that relate to each other and align to a certain context. The four sets of metrics can be found in Appendix S2, with sample questions and methods per set in Appendices S3, S4, S5, and S6. The co‐created sets show some similarities, particularly for input and process metrics, while impact metrics differed widely. The sets can be used as a starting point for M&E by patient engagement initiatives. These sets can also be tailored to each initiative by adding and removing specific metrics to suit the context. However, a tailored set should include metrics related to all components of the framework for a coherent evaluation approach.

## 5 DISCUSSION

To address the need for metrics for M&E of patient engagement in medicine development, the PARADIGM project created a framework with metrics. In this section, we reflect on the framework and its value, looking at four criteria (a) evidence‐base, (b) multi‐stakeholder perceptions, (c) comprehensiveness, and (d) utility. In addition, we reflect on the methodological strengths and limitations of this participatory research project.

### 5.1 Reflections on the framework and its value

The principal strength of the framework is that it is evidence‐informed and iteratively developed with all relevant stakeholders. We integrated literature and existing frameworks^33^, ^34^, ^35^, ^36^ as well as quality guidance documents such as PFMD Quality Guidance,^37^ the TransCelerate Toolkit^38^ and EUPATI guidance^39^ with case studies. We have incorporated dimensions of realist evaluation in order to acknowledge the importance of context and mechanisms in evaluating outcomes.^40^ We developed guidance for metric selection; the relevance and utility of each metric selection criterion varies by setting and user. Some cases mostly selected metrics based on ‘availability of resources’ and ‘alignment with reporting structures and history’ with limited attention to the ‘relevance’, ‘fit with a set’ (coherence with other selected metrics) or 'stakeholder appropriateness’. However, the framework and reflection conversations helped case study participants consider metrics they had not thought of, including those relevant to other stakeholders or to track progress made. The application of the framework requires a multi‐stakeholder approach to ensure that sufficiently diverse metrics are selected.

The M&E framework is comprehensive and intends to facilitate understanding of how patient engagement translates into impact and why initiatives fail or succeed. Besides the conceptual guidance, the framework also provides metrics to operationalize measurement of the different components. The case studies showed that the framework can be applied in multiple therapeutic areas, settings and types of patient engagement initiatives. Although it was developed for taking an engagement initiative as the unit of analysis, some cases also used the framework at an organizational level (for multiple initiatives/studies). Those cases for example measured the number of stakeholders (eg patients, companies) involved in patient engagement activities (input); number and type of patient engagement activities (process); satisfaction level (process); topics or aspects of development plan where patients gave input (learnings); number or percentages of recommendations implemented (changes); perceived added value of patient input (impact); and the number or percentages of projects with patient engagement (impact). The framework could be used at an organizational level; however, a generic set of metrics that can be used at an organizational level has not been created within this project and should be further explored. The selected metrics could be transferable to engagement initiatives in other settings (eg health research or care), but specific (impact) metrics probably require adaptations. However, as Greenhalgh and colleagues found, the relational work involved in planning monitoring and evaluation may be more important than any subsequent framework itself.^22^ Our findings align with this conclusion; the interaction and trust built between actors involved in co‐creating a framework that can be tailored to suit the needs of different groups is likely to be more locally relevant and used than a one‐size‐fits‐all framework. Generating a tailored framework together (eg through a multi‐stakeholder workshop) will influence participants (eg motivation), knowledge (eg incorporation of research, experiential and contextual knowledge) and the process of implementation (eg ownership, testimony of end‐users).^22^

We noticed that some case study participants struggled to understand the linkages between components of the framework, in particular how impacts were triggered, blocked or modified by contextual factors. This suggests that those working in the context may not ‘see’ these factors until an external actor points them out, also known as the cultural phenomenon ‘fish don't know they are in the water’ described by Derek Sivers.^41^ The application of the framework helps to shine a light on the ‘water’. The interpretation of findings requires training and guidance to ensure that contextual factors and metrics relevant to the theory of change are not overlooked. This suggests that applying the framework is a capability that stakeholders will build up over time. Furthermore, the application of the framework may require organizational change, including a broader perspective on what counts as ‘evidence’,^26^ adapted reporting structures, capacity and resources. This requires commitment and two aspects of culture change, for all to embrace:

1. the importance of embedding patient engagement in the development of medicines
2. the philosophy of continuous improvement.

Stakeholders initially preferred to develop purely practical guidance on how to conduct an evaluation, including assessment grids and a ‘fixed’ set of metrics that can be used for benchmarking. To support stakeholders in the relational work that evaluation requires while providing guidance on the reflexive strategy, we developed an adaptive framework with (sets of) metrics that can be tailored to different needs.

A limitation of the framework is the focus on intended objectives, as this may limit the ability to capture unintended effects. Research shows that while engagement brings many benefits, it's also challenging and can lead to some negative consequences (eg delays in study planning, patients feeling undervalued or overburdened).^18^ Having a process for monitoring possible challenges and unintended consequences throughout the engagement process is important for continuous improvement of engagement practices. While accountability and learning were both identified as purposes for evaluation, in practice, evaluations may be more accountability focused to show to what extent objectives are reached. Learning for system innovation requires reflexivity during evaluation.^42^ Ideally, reflexivity becomes accepted practice within a patient engagement initiative through sharing short‐term and intermediate results using informal discussions or methods such as live polling, quizzes, diaries, anonymous survey or feedback forms.^43^

### 5.2 Methodological strengths and limitations

A strength is that we used an iterative multi‐stakeholder approach to create the framework. The framework is in line with other recently proposed metrics^21^, ^44^ though more inclusive in its scope.

Ideally, patient engagement occurs throughout the full medicines’ development cycle, as an on‐going activity. A limitation of our research is the limited number of cases that applied the framework to research priority‐setting or early dialogue decision‐making points as there is less patient engagement in these areas. Therefore, the framework may be more focused on metrics for the decision‐making point related to design of clinical trial. We used literature to complement the case studies and had wide discussions with partners about metrics for other decision‐making points.

The framework, being developed through a multi‐stakeholder approach, is prone to bias in relation to those stakeholders represented in the process. Since patients and regulatory bodies were less well represented than industry, the framework may include more metrics relevant to industry. Stakeholders were mostly Western‐Northern European‐based, and there was limited participation from Central‐Eastern Europe and young people. To correct for this, an additional multi‐stakeholder workshop has been held for these groups to gather their perspectives and any new metrics that derived from this workshop were included in the framework.

The sets of metrics have hardly been tested in practice because very few cases had the opportunity to measure metrics during the project. Therefore, limited insights have been gathered about optimal methods or tools for measurement. However, we created an overview of sample questions, methods and tools drawn from literature and evaluation documents collected which is a starting point for measurement. The framework could also be used to improve the comprehensiveness and rigour of existing measurement tools. The co‐creation of appropriate measurement tools requires further investigation and flexible approaches. Mixed methods and multiple tools are needed as one survey will not be able to capture the complexity and impact of patient engagement.^45^ Longitudinal research is needed in this area as it takes time before impacts become evident.

Further application of the framework is needed to co‐create sets of metrics for different collaborations and contexts. This may result in additional or new proposed metrics and insights. The application of locally relevant frameworks is necessary to better understand in which contexts certain practices lead to valuable patient engagement and why initiatives fail or succeed. To that end, we aim to develop an online interactive tool which enables users to tailor the framework to their situation. This could result in co‐analysis of data gathered by different initiatives and ultimately stimulate continuous improvement of engagement practices and the creation of an evidence library that reinforces the need for a culture shift towards a patient‐centred R&D system.

## 6 CONCLUSIONS

Monitoring and evaluation of patient engagement can enhance meaningful and sustainable patient engagement. The created M&E framework helps to monitor progress and demonstrate impact. There is a large variety in the purposes of M&E and the objectives of patient engagement; accordingly, metrics vary per initiative and stakeholder group. Evaluation studies can help to understand in which contexts certain practices lead to valuable patient engagement and why initiatives fail or succeed. We argue that the value of patient engagement can best be understood by measuring metrics related to all components of the M&E framework using a multi‐stakeholder, reflexive approach.

## CONFLICT OF INTEREST

None declared.

## AUTHOR CONTRIBUTIONS

All authors were part of the metrics working group, they co‐designed and guided the research project and participated in the monthly working group calls. TS and PR co‐led the research project. LV, TF, TS, SF, JB were part of the research team. They collected and analysed the data provided by partners. PR, GB, MB, AD, MD, LE, EF, CH contributed to the case studies by collecting data and contributed to the workshops. All authors were involved the interpretation of data. All authors were involved in drafting the manuscript and approved the final version.

## Data Availability

The data that support the findings of this study are available on request from the corresponding author with permission of the case. The data are not publicly available due to privacy or ethical restrictions. Anonymized data are available in the appendices.
